# How Self-Generated Thought Shapes Mood—The Relation between Mind-Wandering and Mood Depends on the Socio-Temporal Content of Thoughts

**DOI:** 10.1371/journal.pone.0077554

**Published:** 2013-10-23

**Authors:** Florence J. M. Ruby, Jonathan Smallwood, Haakon Engen, Tania Singer

**Affiliations:** 1 Department of Social Neuroscience, Max Planck Institute for Human Cognitive and Brain Sciences, Leipzig, Germany; 2 Department of Psychology, University of York, York, United Kingdom; CSIC-Univ Miguel Hernandez, Spain

## Abstract

Recent work has highlighted that the generation of thoughts unrelated to the current environment may be both a cause and a consequence of unhappiness. The current study used lag analysis to examine whether the relationship between self-generated thought and negative affect depends on the content of the thoughts themselves. We found that the emotional content could strongly predict subsequent mood (e.g. negative thoughts were associated with subsequent negative mood). However, this direct relationship was modulated by the socio-temporal content of the thoughts: thoughts that were past- and other-related were associated with subsequent negative mood, even if current thought content was positive. By contrast, future- and self-related thoughts preceded improvements of mood, even when current thought content was negative. These results highlight the important link between self-generated thought and mood and suggest that the socio-temporal content plays an important role in determining whether an individual's future affective state will be happy or sad.

## Introduction

Thoughts and feelings do not always arise from events in the here and now. Self-generated thoughts (SGT), as reflected by experiences such as mind-wandering and day-dreaming, illustrate that our mind can produce thoughts in a stimulus-independent fashion [1], using previously stored information. Whether their initiation is spontaneous or voluntary, SGT are generated based on intrinsic changes that take place within the individual rather than immediate perceptual input. Studies suggest that these SGT are a core form of human cognition and occupy as much as half of waking mentation [2]–[4].

It is relatively common for SGT to be focused on events that may occur in the future. A prospective bias to SGT is prominent in Europe [2], the USA [3], [5] as well as in China [6] and Japan [7] and content analysis has documented that these future thoughts often involve autobiographical planning [3]. Presumably people use SGT to take advantage of the benefits that prospection affords: they use previously-acquired knowledge to prepare for events that have not yet happened, so that their actions can be more effective if the opportunity to act ever arises [8]–[10].

Consistent with the notion that SGT conveys a long-term benefit, individuals who mind-wander under non-demanding circumstances tend to delay gratification [11] and generate more creative solutions to problems [12]. SGT, however, is not always beneficial and when it occurs during complex tasks such as reading, it is often associated with reduced performance (e.g. [13], [14]). Moreover, in daily life, mind-wandering has been linked to automobile accidents [15]. Evidence of both costs and benefits therefore suggests that SGT is not a homogenous experience [11].

An important negative consequence of SGT emerges through its association with mood. Using experience sampling in more than 2000 participants, Killingsworth and Gilbert [4] observed that episodes of SGT were followed at the next sampling point (hours later or the following day) by lowered mood. Based on the temporal precedence of mind-wandering episodes, they suggested that “mind wandering […] was generally the cause […] of unhappiness”. Similarly, inducing negative mood in participants increases mind-wandering [16] and shifts its temporal focus from the future to the past [17], [18]. In addition, the association between negative affect and past-related thoughts has been documented in individuals with depressive disorders, who excessively ruminate about past failures (e.g. [19], [20]). Together, these results suggest that SGT, especially when focused on the past, may be both the cause and the consequence of negative mood.

Based on their data, Killingsworth & Gilbert (2010) suggest that “a wandering mind is an unhappy mind”, an assumption that would be correct if all types of mind-wandering impacted on mood in a *homogeneous* manner. However, given that SGT can have heterogeneous consequences in other domains (i.e. both costs and benefits), we explored whether its influence on mood might also be heterogeneous. For example, past-related thought may be especially likely to be associated with low mood [17] while other types of thought (e.g. future-focused) may not.

To test these competing hypotheses, we measured mood and SGT in a set of participants while they performed a simple choice reaction time task (CRT). To capture potentially heterogeneous types of SGT, participants answered a series of questions regarding the content of their thoughts i.e. whether they were task-related, focused on different temporal epochs (past or future), involved different referents (self or other) and varied on their emotional tone (positive or negative). Using Principal Component Analysis (PCA), we decomposed these reports based on the patterns of co-variance across different questions, which allowed different types of thoughts to be defined. We then implemented lag analyses using linear mixed models in order to explore the relation between different types of SGT and subsequent mood.

## Methods

### Participants

We recruited 85 German-native speakers from the Max Planck Institute for Human Cognitive and Brain Sciences database. Three participants were excluded as they had an extremely low accuracy on the CRT task. The average age of the remaining participants was 25.5 years (range: 21–31 years) and all had normal or corrected-to-normal vision. Two individuals were left-handed, 35 were females.

### Ethics Statement

The study was approved by the Ethics Commission of the Medical Faculty of the University of Leipzig under the code 360-10-13122010. All the participants gave written consent before the beginning of the experiment and were remunerated 21 Euro for their participation.

### Procedure

#### CRT task

Similar versions of the CRT task have been routinely used in studies on mind-wandering (e.g. [11], [12]).The task lasted 14 min. Stimuli were presented using E-prime 2.0 [21], [22]. Participants observed a sequence of black and colored digits on a computer screen. Only when a colored digit was presented, participants had to indicate whether the digit was odd or even with a button push. Black digits were presented for 1000 ms and colored ones for 2000 ms. Responses had to be made while colored digits were still presented on the screen, or else the trial was considered as missed. Stimuli were separated by a fixation cross of variable duration (2200–4400 ms). Colored and non-colored digits were presented with a ratio of approximately 1/6.

#### Experience Sampling

Intermittently throughout the task, participants were interrupted and instead of being presented with a digit, they were probed about the content of their thoughts. Participants were asked to answer 9 questions using a 9-point Likert scale (see [23], [24] for previous uses of this method). Answers were made using the keyboard and question presentation was self-paced. Participants reported whether their thoughts were related or unrelated to the task, the temporal, social and emotional aspects of the thoughts and their current mood (see **Text S1** for a list of questions). The number of probes and their occurrence were randomly determined (*Mean* number of probes: 7.10, *SE* = .18, *range*: 3–12; *Mean* duration between two probes: 2 min, *SE* = .05 min, *range*: 0.5–8.5 min).

#### Questionnaires

Prior to the experiment, participants completed a battery of online questionnaires including the Beck Depression Inventory (BDI [25]) using the LimeSurvey Tool [26]. The BDI was administered to acquire an established measure of sustained negative affect independent from the mood reports obtained during the laboratory session.

## Results

### Behavioral and Subjective Data

**Figure 1:**
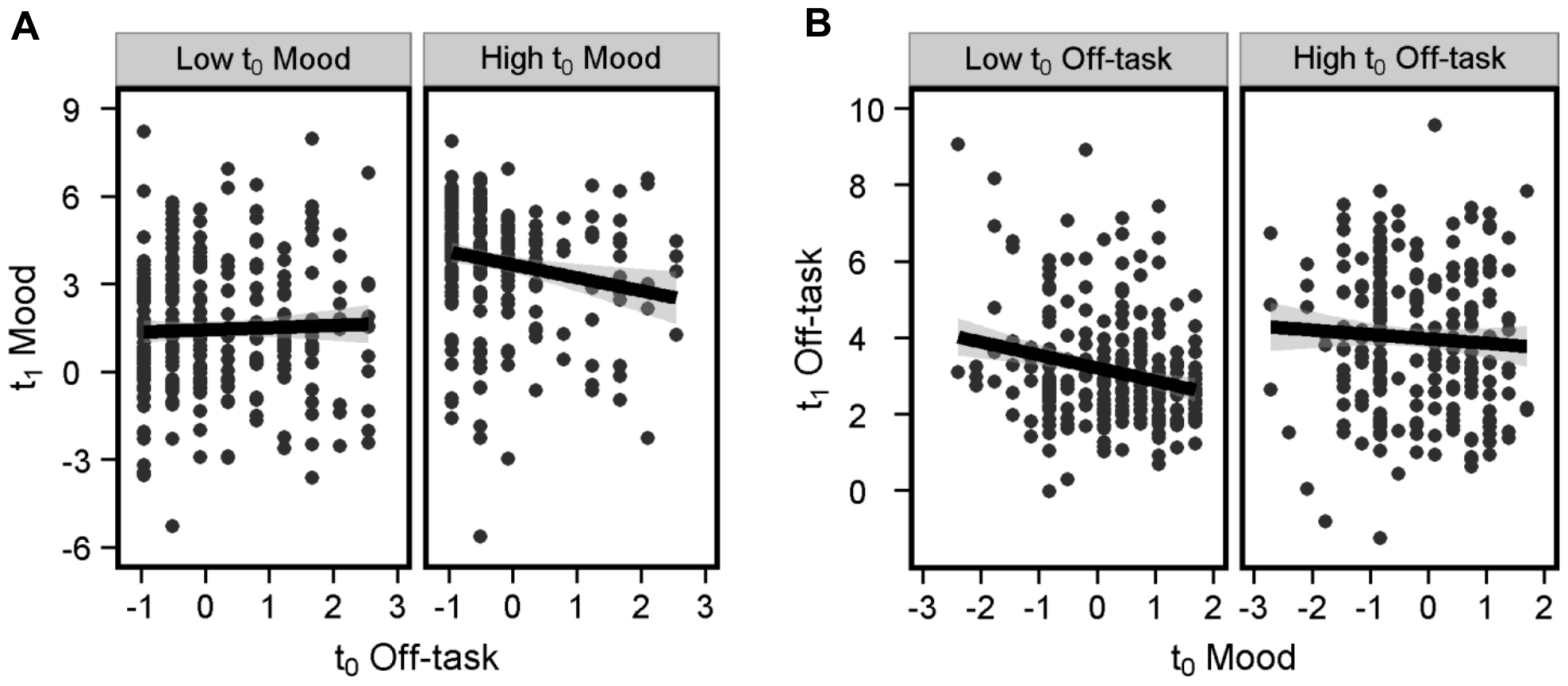
Principal Component Analysis. ***A)*** *Mean probe ratings*. *** indicates t-tests with p values<.001. ***B*** *) Scree Plot from the PCA*, showing the Eigen values of the 7 components obtained. The first three components explained almost 70% of the overall variance in our data. ***C*** *) Heatmap of the 3 Principal Components*, showing the component loadings for each question. The Socio-temporal Past Other Component (ST-PO) weighted positively on Off-task, Other and Past ratings, and marginally on Future ratings. The Affect Component positively weighted on Negative ratings and negatively on Positive ratings (i.e. high scores on this factor reflect more negative SGT). Finally, the ST Future and Self Component (ST-FS) weighted positively on Self, Future and Off-task ratings.

Participants had normal accuracy and response time (RT) during the CRT task (*Mean* accuracy  = .94, *SE* = .01; *Mean* RT = 799.5 ms, *SE* = 14.35). Overall, 590 probes were recorded across 83 participants. For each probe, participants took on average 41 s to answer the 9 questions (mean RT to answer one question: 4531 ms, SE = 71.5 ms). Replicating previous findings (e.g. [2], [3], [17]), SGT were more frequently directed towards the future than the past (paired t-test, t(82) = 5.95, p<.001; **Fig. 1A**). Thoughts were also more frequently self-related than other-related (t(82) = 4.32, p<.001) and rated as more positive than negative (t(82) = 9.39, p<.001), similar to Bernsten and Bohn's findings [27]. The large number of questions that composed the experience sampling procedure may have affected participants' SGT experience. However, the standard performance levels and the presence of the perspective bias in our sample suggest that our experience sampling method was similar to previously reported methods (e.g. [2], [3], [17]).

### Analysis strategy

#### Lag analyses

We used lag-analyses in order to investigate the links between SGT and mood reports. For example, to investigate the link between Off-task ratings and mood, we used Off-task ratings at a given time (e.g. t_0_) to predict the mood ratings of the probe immediately following (e.g. t_1_). This analysis therefore requires that our predicted variable has to be lagged by one period (in this example, the mood ratings). This lagging technique requires that the last probe from every subject has to be discarded (e.g. if the last Off-task rating is used as a t_0_ probe, there is no more mood ratings to use as a t_1_ probe). The lag-analyses reported hereafter are therefore based on data from 507 and not 590 probes.

#### Linear Mixed Models

**Figure 2:**
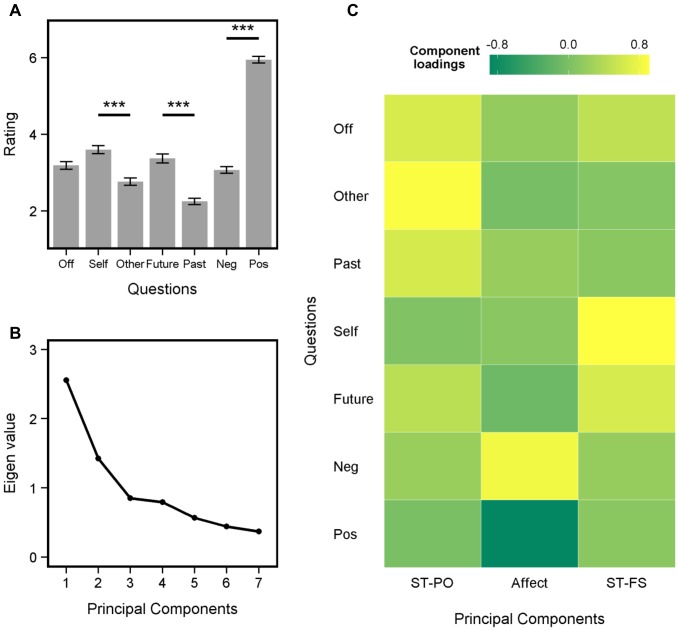
Relation between Off-task ratings and Mood. ***A*** *) Predicting t_1_ Mood from t_0_ Off-task ratings*. When t_0_ Mood was high (right panel), t_0_ off-task focus was linked to a decrease in t_1_ Mood. Data was plotted following a median split on t_0_ Mood. ***B*** *) Predicting t_1_ Off-task ratings from t_0_ Mood*. Negative mood at t_0_ was linked to an increase in t_1_ Off-task, but only when t_0_ Off-task ratings were low (left panel). Data was plotted following a median split on t_0_ Off-task. Thick black lines represent best-fitting linear regressions and gray ribbons represent 95% confidence intervals.

**Figure 3:**
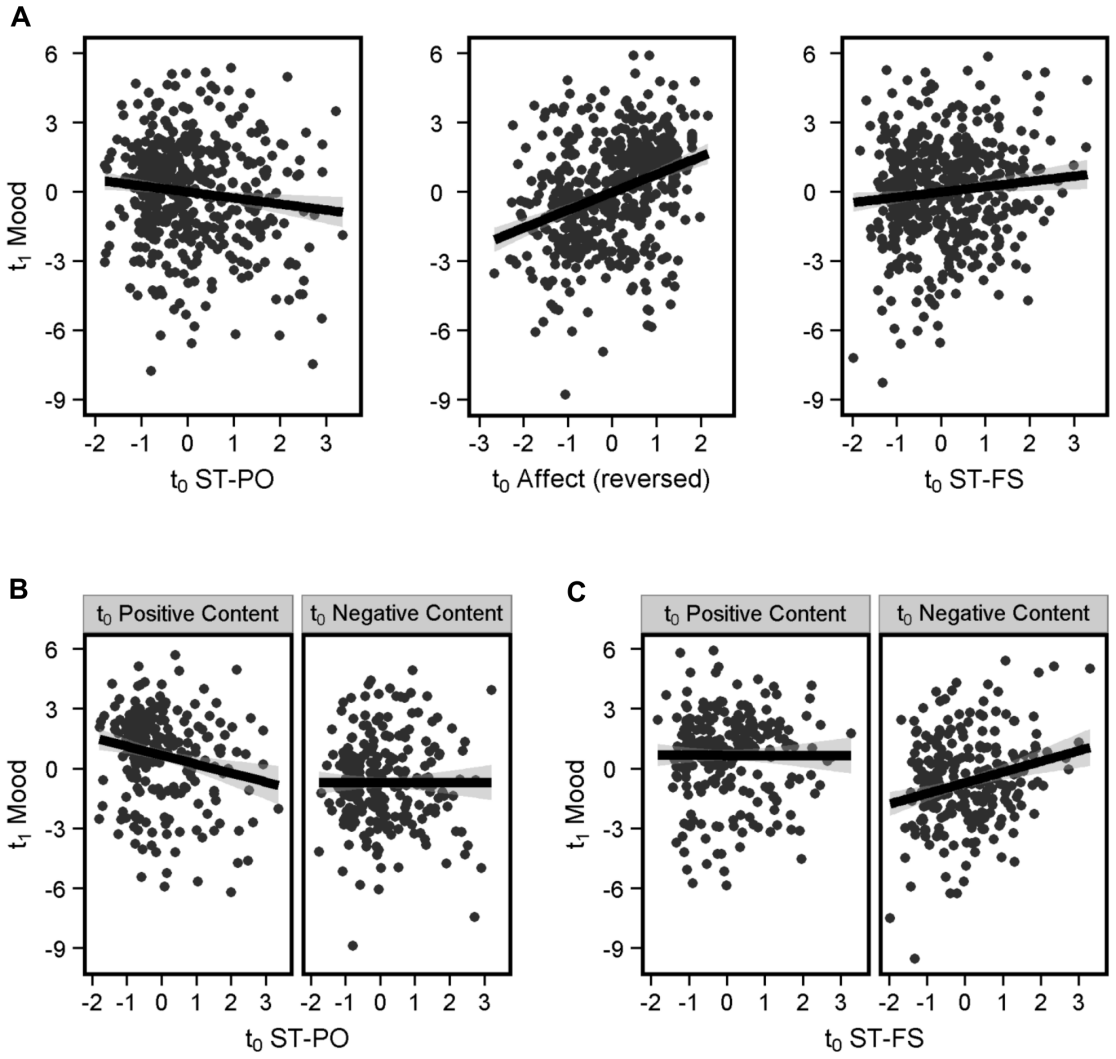
Effect of previous thought content on mood. ***A*** *) Main effects*. All three thought components at t_0_ significantly predicted t_1_ Mood. Increase in t_0_ ST-PO was linked to a decrease in t_1_ Mood (left). Increase in t_0_ positive thoughts was linked to an increase in t_1_ Mood (middle). Please note that for purpose of display, the Affect Component was reversed so that higher values indicate more positive thoughts. Finally, an increase in t_0_ ST-FS was linked to an increase in t_1_ Mood (right). ***B*** *), Interaction between ST-PO and Affect Content*. Especially when thoughts were positive, t_0_ ST-PO was associated with a decrease in t_1_ Mood. Data was plotted following a median split on t_0_ Affect Component. ***C*** *). Interaction between ST-FS and Affect Content*. Especially when thought content was negative, t_0_ ST-FS was associated with an increase in t_1_ Mood. Thick black lines represent best-fitting linear regressions and gray ribbons represent 95% confidence intervals.

Linear mixed models (LMMs, [28]) were used to perform lag analyses as they allow to estimate both fixed effects (effects that one is interested in e.g. the effect of t_0_ Off-task ratings on t_1_ Mood) and random effects (effects that arise due to groups within the data e.g. multiple sampling within a subject). All predictor variables (but not dependent variables) were z-transformed prior to performing lag analyses. All LMMs included one random effect (the intercept for each Subject) to control for the dependency arising due to repeated sampling of data within subjects. Time of t_1_ probe onset was included as a fixed effect to account for the fact that mood is likely to decrease as the task continues. Finally, for LMMs predicting t_1_ Mood, t_0_ Mood was also included in the analysis as a fixed effect in order to control for the auto-correlation between t_0_ and t_1_ Mood. Data were analyzed and plotted using R.15.2, lme4, languageR and ggplot2 packages [29]–[32]. Because of the large size of our data set and because we only estimate a single random effect the significance of the fixed effects can be estimated using Markov chain Monte Carlo methods, via the *pvals.fnc* function provided in languageR package [29]. Before plotting a dependent variable as a function of a single predictor (e.g. **Fig. 2** and **Fig. 3**), it was adjusted for all the other predictors specified in the model. Median splits were used to visualize results when significant interactions were obtained.

### Relation between off-task thinking and mood

First, we aimed to replicate the findings that SGT can both precede and follow unhappy mood [4], [17]. We conducted a lag-analysis predicting t_1_ Mood from t_0_ Off-task ratings, including t_0_ Mood in the analysis. This revealed a significant interaction between t_0_ Off-task and t_0_ Mood (See **Table 1** for a full description of the results). As seen in **Fig. 2A**, there was a negative relation between t_0_ Off-task and t_1_ Mood especially when t_0_ Mood was positive (right panel). This suggested that off-task thinking preceded decreases in t_1_ Mood, especially when t_0_ Mood was positive. A second lag-analysis (predicting t_1_ Off-task from t_0_ Mood) also revealed a negative relation between t_0_ Mood and t_1_ Off-task ratings, especially when t_0_ Off-task ratings were low (**Table 2** and **Fig. 2B**, left panel). This suggests that low t_0_ Mood was associated with high t_1_ Off-task ratings, especially when t_0_ Off-task ratings were low.

**Table 1:** Results of the LMM lag-analysis predicting t_1_ Mood from t_0_ Off-task.

	Coefficient	SE	t value	p-value
Mean t_1_ Mood	2.432	0.182	13.38	<0.001
t_1_ time of probe onset	−0.199	0.108	−1.85	0.0765
t_0_ Off-task	−0.040	0.131	−0.307	0.759
t_0_ Mood	1.355	0.137	9.86	<0.001
t_0_ Off-task x t_0_ Mood	−0.218	0.111	−1.97	0.050

**Table 2:** Results of the LMM lag-analysis predicting t_1_ Off-task from t_0_ Mood.

	Coefficient	SE	t value	p-value
Mean t_1_ Off-task	3.608	0.161	22.35	<0.001
t_1_ time of probe onset	0.372	0.085	4.37	<0.001
t_0_ Mood	−0.224	0.112	−2.00	0.046
t_0_ Off-task	0.474	0.106	4.48	<0.001
t_0_ Mood x t_0_ Off-task	0.200	0.088	2.26	0.024

These results broadly replicate previous studies: i) SGT followed negative mood [16]–[18] and ii) SGT preceded negative mood, although only when previous mood was positive [4]. We suspect that t_0_ Off-task did not significantly predict t_1_ Mood when t_0_ Mood was negative (**Fig. 2A**, left panel) because of the auto-correlation of mood between t_0_ and t_1_ (r = 0.70, p<.001). In other words, only in the case where t_0_ Mood is positive can the negative effect of t_0_ Off-task be dissociated from the autoregressive properties of mood.

### Role of thought content in the relation between mind-wandering and mood

#### PCA Analysis

Next, we explored the role of content in the relationship between negative mood and SGT. PCA with Varimax rotation was used to decompose the 9 probe questions based on the co-variance within our data. Three Principal components were obtained, explaining nearly 70% of the total variance (**Fig. 1B and 1C**). We obtained two socio-temporal (ST) factors and a single affective factor: 1) A Past and Other Component (ST-PO), weighting strongly on past, other and Off-task ratings. 2) An Affect Component, weighting strongly on the negative and positive dimensions (because the Affect component *positively* weights on *negative* ratings, high scores imply negative SGT). 3) A Future and Self Component (ST-FS), weighting on future, self and Off-task ratings.

#### Lag analysis

Lag analysis was used to examine whether the socio-temporal or the emotional content of SGT was linked to subsequent mood. In this analysis, t_1_ Mood was our dependent variable and the three t_0_ PCA factors were independent variables. The analysis revealed five significant effects (**Table 3**). i) An effect of the Affect Component, suggesting that positive thought was associated with positive mood at the next probe (**Fig. 3A**, middle); ii) An effect of ST-PO, indicating that this type of socio-temporal content was associated with a subsequent reduction in mood (**Fig. 3A**, left); iii) An interaction between ST-PO and Affect Component (**Fig. 3B**), indicating a negative relation between ST-PO and mood *even* if the current content of thought was positive; iv) A main effect of ST-FS, indicating that this type of socio-temporal thought was linked to an improvement of subsequent mood (**Fig. 3A**, right); v), An interaction between the ST-FS and Affect Components, indicating that the positive relation between mood and future and self thinking was more pronounced when the current content of thought was negative (**Fig. 3C**). As in the analysis with Off-task ratings, the strong relation between t_0_ Affect component and subsequent mood is likely to partially mask the effects of ST components. Overall, these results suggest that the relation between SGT and mood depends on its content.

**Table 3:** Results of the LMM lag-analysis predicting t_1_ Mood from t_0_ SGT components.

	Coefficient	SE	t value	p-value
Mean t_1_ Mood	2.509	0.136	18.52	<0.001
t_1_ time of probe onset	−0.131	0.106	−1.24	0.215
t_0_ Mood	1.27	0.161	7.91	<0.001
t_0_ ST-PO	−0.262	0.117	−2.24	0.026
t_0_ Affect Component	−0.773	0.159	−4.87	<0.001
t_0_ ST-FS	0.229	0.114	2.00	0.046
t_0_ ST-PO x t_0_ Affect Component	0.366	0.105	3.49	<0.001
t_0_ Affect Component x t_0_ ST-FS	0.319	0.116	2.74	0.006
t_0_ ST-PO x t_0_ ST-FS	0.131	0.110	1.19	0.233
t_0_ ST-PO x t_0_ Affect Component x t_0_ ST-FS	0.201	0.120	1.68	0.094

#### Controlling for co-linearity between Emotional Content and Mood

Mood and Affect Component were highly correlated, both at t_0_ and between t_0_ and t_1_ lags (**Table 4**). To understand whether this co-linearity might have been responsible for the heterogeneous effects of SGT types, we calculated the difference in Mood between t_0_ and t_1_ lags (Mood_Diff_). Using a similar lag analysis as before (see **Table S1**) we found comparable results: ST-PO accompanied by positive content was associated with an increase in negative mood (**Fig. S1A**), while ST-FS was linked to increases in mood, especially when participants experienced negative thoughts (**Fig. S1B**). In this analysis, the correlation between t_0_ Affect component and Mood_Diff_ was substantially reduced, suggesting that co-linearity in our initial model cannot be responsible for the heterogeneous effects of thought types on mood.

**Table 4:** Pearson's correlations between t_0_ Affect Component and mood measures.

	t_0_ Affect Component	
	r	p-value
t_0_ Mood	−0.72	<.001
t_1_ Mood	−0.59	<.001
Mood Diff	0.13	.003

#### Link to BDI Score

Finally, we investigated whether the content of SGT could predict the BDI. Replicating previous studies indicating that past-related thought is a characteristic of dysphoria [17], we found that past- other-related thoughts as well as negative thoughts were associated with higher BDI scores (Univariate ANOVA predicting logged BDI score from PCA Components; main effects, ST-PO: F = 4.30, p = .04; Affect Component: F = 3.62, p = .06). By contrast, there was no effect of ST-FS Component (F = 0.27, p>.6). This demonstrates that an established measure of chronic low mood is accompanied by SGT directed towards the past rather than the future.

## Discussion

Our study examined whether the relation between SGT and negative mood depends on the content of thought. Using experience sampling during a short undemanding task, we replicated the findings that SGT can both precede and follow negative mood [4], [16]–[18]. However, we found that this relationship depends on the content of thought. Past- other-related thoughts were linked to decreases in mood, even if SGT content was positive. By contrast, SGT focused on the future and self was linked to increases in positive mood, even if the content of thought was negative. As the most pronounced impact of a future focus on mood was found when thoughts were negative, our data underline that unhappiness and SGT are inextricably linked. However, the opposing effects of ST-PO and ST-FS components demonstrate that the occurrence of certain kinds of SGT may constrain rather than prolong negative mood.

However, the correlational design of our study does not allow us to conclude that certain types of SGT *caused* subsequent decrease or increase in mood. In order to establish a causal link between SGT and mood, future research will have to provide specific models that explain the possible mechanisms that may allow SGT to directly influence mood. For example, it has recently been proposed that it is necessary to distinguish between influences that determine the occurrence of SGT and those that control the continuity of the experience once initiated [1]. In this context, because our results indicate a link between past-other-related SGT and negative mood, it raises the possibility that the reprocessing of past events may initiate a cyclical process that underlies the link between SGT and unhappiness. In this regard our study remains informative as it reveals that only certain but not all types of SGT have a negative relationship to mood, an observation which provides a boundary condition on more specified models in the future.

Although we replicated the findings that SGT can precede negative mood, this was only the case when t_0_ Mood was positive. As mentioned earlier, this may be caused by a ceiling effect (the negative effect of SGT may only be observed when t_0_ Mood is not too low) but it may also be caused by a regression to the mean i.e. high t_0_ Mood may be linked with high t_0_ positive Off-task thoughts. However at t_1_, the Off-task thoughts may no longer be as positive, leading to a decrease in mood back to average levels. Even though this effect may explain the link between Off-task and Mood, regression to the mean cannot account for the opposing effect of ST-PO and ST-FS as the emotional content was included in the corresponding lag analyses. For example, positive emotional content at t_0_, in the absence of ST-PO, did not lead to less positive mood.

The conditions under which SGT were recorded may also influence our conclusions. Following studies showing that SGT are most common during non-demanding situations (e.g. [11], [33]), we measured SGT during an easy CRT task which requires limited cognitive resources to be performed properly. The task therefore puts individuals into a non-demanding situation and allows them to frequently generate SGT if desired. In addition, based on studies showing similarities between SGT under laboratory conditions and in everyday life (e.g. [34]), we suppose that our results are likely to generalize to daily life especially under circumstances where current perceptual input is not especially salient (e.g. while being stuck in a traffic jam).

In addition to demonstrating the heterogeneity of the relation between SGT and mood, our study also demonstrates that SGT can be informatively characterized according to the covariation between distinct constituents of the content. Our application of PCA revealed a statistical overlap between self-related and future-related thoughts that corroborates prior work. For example, a brief period of self-reflection caused an increase in future-related thoughts [35]. In another study, ratings of open-ended reports of SGT revealed that future-related thought were also highly self-related [3]. The PCA also grouped together the past and other constituents into one component which was associated with negative mood. This statistical characterization of SGT is consistent with prior work [17] linking past-related thought to negative affect both at a transient level (i.e. following mood induction) and more sustained level (i.e. BDI). The fact that PCA revealed statistical categories of SGT whose psychological properties mimic the results of experimental manipulations provides independent support for the existence of psychologically distinct types of SGT.

Finally, the *current concerns hypothesis* [36], [37] provides a valuable perspective on the observation that past and future-related SGT have opposing links to mood. According to this framework, mind-wandering often arises because unfulfilled goals or ambitions have greater salience than current environmental inputs (see also [1]). If individuals simply simulated a current concern, without attempting to generate possible solutions, this could prolong the influence that the unfulfilled goal has on mood. By contrast, future-related thoughts allow individuals to create plans [3], [9] and so could provide the individual with mental strategies or heuristics that could then be used to resolve these issues in the future (e.g. [10]). In this way self and future-related thoughts may reduce the negative influence that current concerns have on mood. Although the capacity to limit mind-wandering may be the best way to improve happiness in the long run, the strong tendency of the mind to spontaneously generate thoughts may hinder this possibility. Our data suggests that, if mentally setting aside a problem is not an option, moving forward by adopting a future focus may be the next best strategy.

## Supporting Information

Figure S1 **Effect of thought content on mood change.** **A**) t_0_ ST-PO was associated with a negative change of mood, especially when t_0_ thought content was positive. **B**) t_0_ ST-FS was linked to a positive change of mood, especially when t_0_ thought content was negative.
(TIF)

Table S1 **LMM predicting mood change from previous thought content.** Fixed effects estimates for the linear mixed model predicting Mood_Diff_ (i.e. the difference between t_1_ Mood and t_0_ Mood) from t_0_ content. We included the Subject as a random effect.
(DOCX)

Text S1 **Thought sampling questions.** The following 9 questions were presented during the thought sampling procedure. Participants responded using a 9-point Likert scale (1: not at all, 9: completely). For the off-task question, 1 indicated “*I was thinking exclusively about the task*” and 9 indicated “*I wasn’t thinking at all about the task*”.
(DOCX)
