# Supplementary figures and images for: How Self-Generated Thought Shapes Mood—The Relation between Mind-Wandering and Mood Depends on the Socio-Temporal Content of Thoughts
Source: PLoS One. 2013 Oct 23;8(10):e77554. doi: 10.1371/journal.pone.0077554 (PMC3806791; doi:10.1371/journal.pone.0077554)

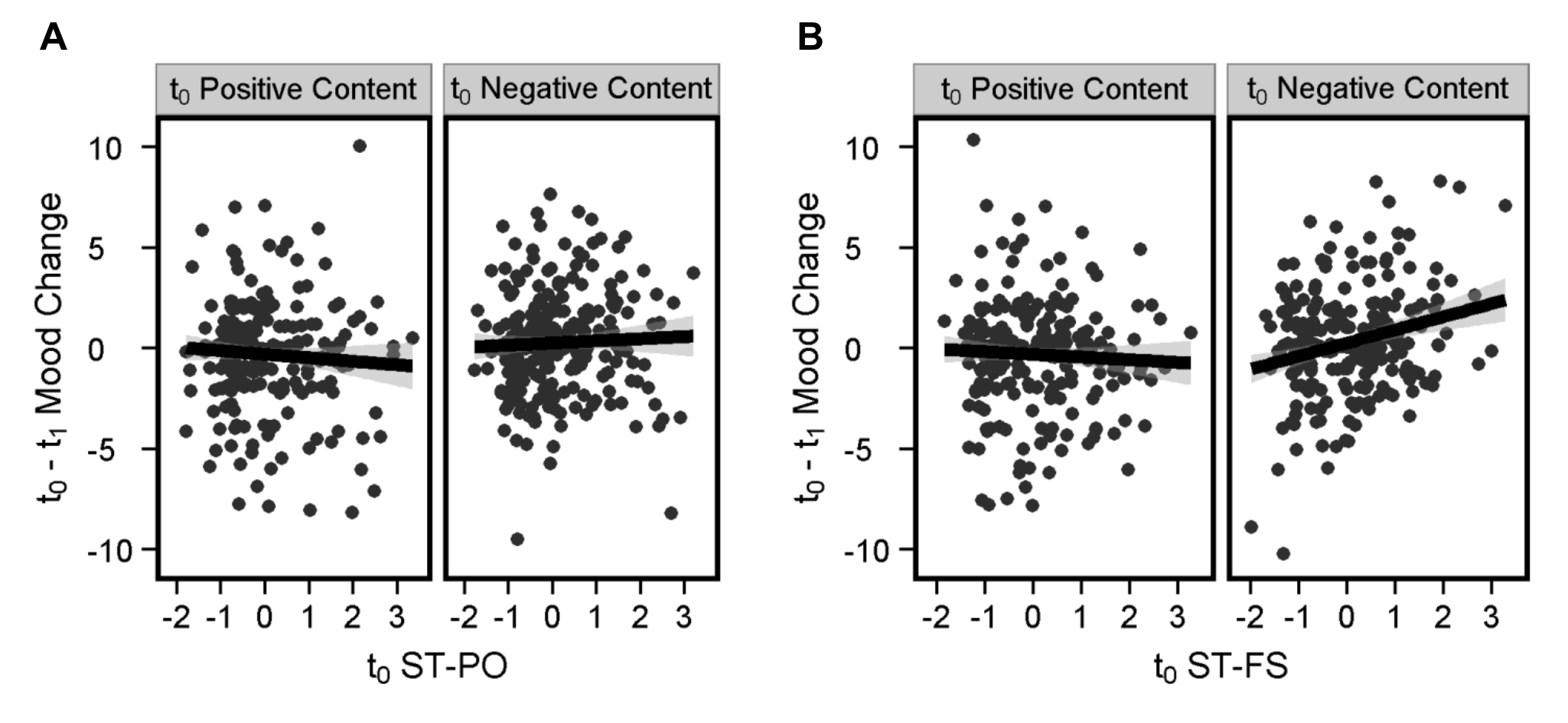

Supplement: Figure S1 — Effect of thought content on mood change. A) t0 ST-PO was associated with a negative change of mood, especially when t0 thought content was positive. B) t0 ST-FS was linked to a positive change of mood, especially when t0 thought content was negative. (TIF) [file pone.0077554.s001.tif]
