# Supplementary material for: How Self-Generated Thought Shapes Mood—The Relation between Mind-Wandering and Mood Depends on the Socio-Temporal Content of Thoughts
Source: PLoS One. 2013 Oct 23;8(10):e77554. doi: 10.1371/journal.pone.0077554 (PMC3806791; doi:10.1371/journal.pone.0077554)
Supplement: Table S1 — LMM predicting mood change from previous thought content. Fixed effects estimates for the linear mixed model predicting MoodDiff (i.e. the difference between t1 Mood and t0 Mood) from t0 content. We included the Subject as a random effect. (DOCX) [file pone.0077554.s002.docx]

**Table S1. LMM predicting mood change from previous thought content.**

|  | Coefficient | SE | t value | p-value |
| --- | --- | --- | --- | --- |
| Mean Mood_Diff_ | -0.110 | 0.114 | -0.97 | 0.333 |
| t1 time of probe onset | -0.134 | 0.118 | -1.14 | 0.257 |
| t0 ST-PO | -0.031 | 0.118 | -0.26 | 0.791 |
| t0 Affect Component | 0.337 | 0.114 | 2.94 | 0.003 |
| t0 ST-FS | 0.235 | 0.115 | 2.04 | 0.042 |
| t0 ST-PO x t0 Affect Component | 0.265 | 0.110 | 2.42 | 0.016 |
| t0 Affect Component x t0 ST-FS | 0.433 | 0.121 | 3.57 | < 0.001 |
| t0 ST-PO x t0 ST-FS | 0.110 | 0.118 | 0.93 | 0.353 |
| t0 ST-PO x t0 Affect Component x t0 ST-FS | 0.262 | 0.131 | 2.01 | 0.045 |

Fixed effects estimates for the linear mixed model predicting Mood_Diff_ (i.e. the difference between t_1_ Mood and t_0_ Mood) from t_0_ content. We included the Subject as a random effect.
