# Supplementary material for: How Self-Generated Thought Shapes Mood—The Relation between Mind-Wandering and Mood Depends on the Socio-Temporal Content of Thoughts
Source: PLoS One. 2013 Oct 23;8(10):e77554. doi: 10.1371/journal.pone.0077554 (PMC3806791; doi:10.1371/journal.pone.0077554)
Supplement: Text S1 — Thought sampling questions. The following 9 questions were presented during the thought sampling procedure. Participants responded using a 9-point Likert scale (1: not at all, 9: completely). For the off-task question, 1 indicated “I was thinking exclusively about the task” and 9 indicated “I wasn’t thinking at all about the task”. (DOCX) [file pone.0077554.s003.docx]

**Text S1. Thought sampling questions.** The following 9 questions were presented during the thought sampling procedure. Participants responded using a 9-point Likert scale (1: not at all, 9: completely). For the off-task question, 1 indicated “*I was thinking exclusively about the task*” and 9 indicated “*I wasn’t thinking at all about the task*”.

*Thought-related questions*:

Off-task: Were you thinking about the task or about something else?
Other: How much were you thinking about other people?

Past: How much were you thinking about the past?
Self: How much were you thinking about yourself?

Future: How much were you thinking about the future?
Pos: How positive were your thoughts?

Neg: How negative were your thoughts?

*Mood-related questions*:

Pos: How positive was your mood?
Neg: How negative was your mood?
